# Dynamics of anterior pituitary function in the acute phase of traumatic brain injury: a prospective cohort

**DOI:** 10.62675/2965-2774.20250106

**Published:** 2025-09-25

**Authors:** Eder Cáceres, Juan Olivella-Gómez, André Emilio Viñán Garcés, Paula Oriana Narvaez-Ramirez, Saber Zafarshamspou, Chad Cole, Archana Hinduja, Afshin A. Divani, Luis Felipe Reyes

**Affiliations:** 1 Unisabana Center for Translational Science School of Medicine Universidad de La Sabana Chía Colombia Unisabana Center for Translational Science, School of Medicine, Universidad de La Sabana - Chía, Colombia.; 2 School of Engineering Universidad de La Sabana Chía Colombia School of Engineering, Universidad de La Sabana - Chía, Colombia.; 3 Department of Critical Care Clínica Universidad de La Sabana Chía Colombia Department of Critical Care, Clínica Universidad de La Sabana - Chía, Colombia.; 4 Department of Surgery School of Medicine Rafsanjan University of Medical Sciences Rafshanjan Kerman Iran Department of Surgery, School of Medicine, Rafsanjan University of Medical Sciences - Rafshanjan, Kerman, Iran.; 5 Department of Neurology The Ohio State University Columbus Ohio United States Department of Neurology, The Ohio State University - Columbus, Ohio, United States.; 6 Department of Neurology The University of New Mexico Albuquerque New Mexico United States Department of Neurology, The University of New Mexico - Albuquerque, New Mexico, United States.; 7 Department of Neurosurgery The University of New Mexico Albuquerque New Mexico United States Department of Neurosurgery, The University of New Mexico - Albuquerque, New Mexico, United States.; 8 Pandemic Sciences Institute University of Oxford Oxford United Kingdom Pandemic Sciences Institute, University of Oxford - Oxford, United Kingdom.

**Keywords:** Brain injuries, traumatic, Hypophysis, Pituitary gland, Pituitary diseases, Intensive care

## Abstract

**Objective:**

Traumatic brain injury leads to disruption of the hypothalamic-pituitary axis. The aim of this study was to evaluate anterior pituitary gland function in the acute phase following traumatic brain injury and its relationship with patient outcomes.

**Methods:**

This was a prospective cohort of traumatic brain injury patients admitted to the intensive care unit. The levels of adrenocorticotropic hormone, growth hormone, and thyroid-stimulating hormone on Days 0, 3 and 7 after the injury were measured. The Glasgow Outcome Scale-Extended (GOSE) was used for 6-month outcomes.

**Results:**

A total of 88 traumatic brain injury patients (79% male, 41 ± 19 years old) who were admitted to the intensive care unit were studied. The frequencies of hormone levels below the range were as follows: adrenocorticotropic hormone, 81% on Day 0, 75% on Day 3, and 68% on Day 7; growth hormone, 76% on Day 0, 65% on Day 3, and 61% on Day 7; and thyroid-stimulating hormone, 42% on Day 0, 41% on Day 3, and 14% on Day 7. Traumatic brain injury severity was associated with Day 0 adrenocorticotropic hormone (p = 0.03) and Day 7 growth hormone (p = 0.03) levels and inversely associated with Day 3 thyroid-stimulating hormone (p = 0.03) levels. Glial fibrillary astrocytic protein was directly associated with Day 3 adrenocorticotropic hormone (OR 1.02, 95%CI 1.01 - 1.03; p < 0.001) and inversely associated with Day 7 thyroid-stimulating hormone (OR 1.02, 95%CI: 1.02 - 1.03; p = 0.04) levels. There was no significant association between hormone levels and mortality or the 6-month Glasgow Outcome Scale-Extended score.

**Conclusion:**

Anterior pituitary hormone disturbances are common following a traumatic brain injury, and the degree of dysfunction is related to the injury severity. No associations were found with mortality or disability. Further investigations are warranted to standardize the measurement of pituitary function after traumatic brain injury and clarify its prognostic/therapeutic role.

## INTRODUCTION

Traumatic brain injury (TBI) is one of the most common neurological injuries and a leading cause of disability and fatality worldwide.^(1)^ The central nervous system maintains continuous bidirectional communication with extracranial organs,^(2-4)^ and TBI is frequently associated with systemic complications.^(5-8)^

The hypothalamic-pituitary axis is crucial for homeostasis in the body, and its dysfunction can present with a wide variety of unspecific clinical signs and symptoms that are not readily identifiable.^(8)^ In the case of TBI, the pituitary gland or the hypothalamic–pituitary area can be affected directly, leading to transection of the pituitary stalk and damage to the hypophyseal portal veins, or can be affected by secondary injury (ischemia, inflammation, excitotoxicity).^(9)^ Although changes in pituitary function might be adaptive to acute injury due to fasting and inflammation in critically ill patients, direct injury to the pituitary gland makes the interpretation of these findings more complicated.^(10,11)^ Autopsy reports show that the most common abnormality in patients who die in the acute phase of TBI is anterior lobe infarction, which might be associated with hemorrhage and necrosis (26 - 86%).^(12-14)^ Magnetic resonance imaging studies of the pituitary gland after a TBI have revealed structural abnormalities, including partial transection of the infundibular stalk and hemorrhage.^(15-17)^ Some potential predictors of anterior pituitary dysfunction, such as TBI severity, age, and skull fractures, have been identified, although these findings have not been consistent across studies.^(18,19)^ Endocrine disorders might not be associated with mortality; however, they are treatable causes of morbidity, and their identification improves recovery and quality of life.^(17,18,20)^ In addition, endocrine disorders might contribute to the development of chronic conditions, such as diabetes mellitus^(21)^ and cardiovascular disease.^(22)^ A frequent challenge encountered when evaluating pituitary axis function is heterogeneity in the time at which the evaluation is made and the method used; some studies have used different stimulation tests in the evaluation of growth hormone (GH) and adrenocorticotropic hormone (ACTH) levels, whereas others have not.^(16,20,23)^ Guidelines recommend pituitary function screening 3 - 6 months after the injury for TBI patients hospitalized > 48 hours, especially if they have symptoms consistent with hypopituitarism, such as fatigue, mood disorders, weight changes, difficulty concentrating, postural hypotension, dizziness and/or sexual dysfunction.^(23,24)^ However, these symptoms can often be attributed to the trauma itself or other associated conditions.

Information on the dynamics of anterior pituitary hormone levels in the acute stage after moderate–severe TBI, as well as its clinical consequences, is limited, with a lack of consensus on the method and time of measurement.^(25)^ Previous studies reported at least one anterior pituitary hormone alteration in 10% - 50% of TBI patients within the first week.^(26-28)^ Nevertheless, less information is available regarding its clinical consequences, which is crucial for determining which patients might need closer follow-up. In general, although the body of literature on hypopituitarism after TBI has been growing, there is still a need to increase awareness and better understand the clinical repercussions of this pathology.^(19)^ This study aimed to describe the frequency of anterior pituitary disturbance in the first week after TBI and its associations with mortality and disability.

## METHODS

The Institutional Review Board/Independent Ethics Committee (IRB/EC) approved the study under local regulations and the Declaration of Helsinki for clinical practices, including obtaining informed consent from the patient representative. All clinical data were anonymized and collected using the Research Electronic Data Capture (REDCap^®^), which was provided by the Universidad de La Sabana.

### Study population

This single-center prospective cohort study was conducted at the Universidad de La Sabana Neurotrauma Center in Chía, Colombia. We collected data from patients with moderate–severe TBI admitted to the neuro-ICU from December 2019 to December 2022.^(29)^ The study included ≥ 18-year-old TBI patients admitted to the neuro-ICU within 24 hours after the injury and who stayed in the neuro-ICU for more than 48 hours. Patients with a previous history of disability or debilitating diseases measured by a modified Rankin scale (mRS) > 2 and those admitted after 24 hours or longer after injury were excluded.

### Definitions

To evaluate the severity of TBI, we used the Glasgow Coma Scale (GCS) and the Abbreviated Injury scale (AIS) of the head (head-AIS). The AIS incorporates both clinical and imaging findings,^(30,31)^ enabling a more nuanced assessment of the severity of the lesion and providing a robust correlation with outcomes. The AIS score can be classified as 1 (minor injury), 2 (moderate), 3 (serious), 4 (severe), 5 (critical) or 6 (fatal). To assess the severity of the overall trauma, we used the injury severity score (ISS), a composite measure derived from the AIS score that includes a rating of the three most severely injured body regions and ranges from 0 to 75. An ISS of 15 or higher is considered major trauma.^(31)^ The AIS ranks injuries in each body region on an ordinal scale from 0 to 6 (from no injury to fatal).^(32,33)^

To characterize the severity of the brain injury in a head computed tomography (CT) scan, we used the Marshall classification, which uses six categories (I - VI) of increasing severity on the basis of noncontrast CT scan findings, including midline shift, compression of cisterns, and mass lesions.^(34,35)^ The glial fibrillary astrocytic protein (GFAP) level was also assessed on admission as a potential diagnostic and prognostic biomarker in acute brain injury.^(36)^

To evaluate mortality and disability as outcomes, we selected the Glasgow Outcome Scale-Extended (GOSE), an ordinal scale of eight points ranging from death to good recovery.^(37-40)^ Trained staff administered the GOSE through a standardized phone interview with the patients or their caregivers 6 months after the injury. For the analysis, we dichotomized GOSE scores into favorable and unfavorable outcomes. A favorable outcome (GOSE score > 4) was considered for those with upper severe disability to upper good recovery, and an unfavorable outcome was defined as a lower severe disability to death (GOSE score ≤ 4).

Blood samples (drawn between 8 and 9 AM) were collected on admission (within 24 hours after the injury, Day 0) and on Day 3 and Day 7 after the injury. We measured the serum levels of ACTH, thyroid-stimulating hormone (TSH), and GH using the multiplex immunoassay MILLIPLEX^®^ Luminex Technology (Merck KGaA, Darmstadt, Germany) for the three collected time-point samples.

### Data collection

Upon admission to the neuro-ICU, demographic data, trauma severity, and prognostication scales were recorded prospectively. Vital signs and laboratory tests were reviewed and confirmed directly from the electronic medical records. At the 6-month follow-up, patients or their legal representatives were contacted via phone by a trained research team member for completion of the GOSE.

### Statistical analysis

Continuous variables were summarized on the basis of clinical relevance and distribution using minimum and maximum values, means ± standard deviations (SDs) for measurements with a normal distribution, or medians and interquartile ranges (IQRs) for measurements without a normal distribution (determined using the Shapiro‒Wilk test for normality). Dichotomous variables are presented as the frequencies and percentages. Differences in variability were evaluated using ANOVA. Differences between intervention groups were assessed via the chi-square test or Fisher’s exact test for categorical variables. In contrast, continuous variables were evaluated using Student’s t test or the Mann‒Whitney U test, depending on their distribution.

A multivariate logistic regression model was constructed for the general cohort to investigate the risk factors associated with unfavorable outcomes at the 6-month follow-up. The model was adjusted for admission demographic data, vital signs, and laboratory tests. The logistic regression used the best subset method for variable selection and included variables with a p value < 0.10 in the univariate analysis. Odds ratios (ORs) with 95% confidence intervals (95%CIs) were calculated on the basis of the exponential values of the coefficients obtained from the final model D. We used R Studio (version 2023.09.1+494) for the analysis.

## RESULTS

During the study period, we collected information from 88 TBI patients admitted to the neuro-ICU (79% [70] male and 41 ± 19 years old). The demographic data, severity of injury and results of laboratory analysis, including admission blood tests, are presented in table 1.

**Table 1:** Demographic characteristics of the traumatic brain injury population

Characteristics	Overall (n = 88)
Age	41 ± 19
Sex, male	70 (79)
Causes of trauma	
Motor vehicle accident	47 (53)
Falls	22 (25)
Cycling	11 (13)
Violence	4 (5)
Glasgow coma scale	8 (6 - 12)
Head-AIS	4 (3 - 4)
Head-AIS	
2 - Moderate	13 (14)
3 - Serious	24 (28)
4 - Severe	40 (45)
5 - Critical	11 (13)
Marshall CT classification	
Diffuse injury I	24 (27)
Diffuse injury II	38 (43)
Diffuse injury III	9 (10)
Evacuated mass lesion	17 (20)
Admission ISS	26 (17 - 34)
APACHE II	14 ± 7
SOFA	7 ± 3
Charlson	0
Admission MAP	87 (77 - 95)
Primary injury on CT	
Epidural hematoma	55 (62)
Contusion	52 (59)
tSAH	45 (51)
Subdural hematoma	27 (30)
Admission tests	
WBC (x 10^3^/dL)	16,0 (12 - 20)
Neutrophils (%)	82 (75 - 88)
Hemoglobin (g/dL)	14.1 (12.35 - 15.1)
Platelet count (x 10^3^/uL)	230.4 ± 81
Serum glucose (g/dL)	130 (110 - 162)
Creatinine (mg/dL)	0.9 (0.7 - 1.1)
Sodium(mEq/L)	140 (137 - 142)
pH on ABG	7.34 (7.2 - 7.41)
PaO_2_ (mmHg)	88 (71 - 126)
PaCO_2_ (mmHg)	35 (30 - 41)
ABG bicarbonate (mmHg)	18.8 ± 3.5
Lactate (mmol/L)	2.6 (1.7 - 3.7)
GFAP (ng/mL)	1.0 (0.2 - 254)
Hospitalization	
Neuro-ICU LOS (days)	6 (4 - 15)
Total LOS (days)	11 (7 - 29)
6-month GOSE	
1	25 (285)
2	2 (12)
3	9 (10)
4	8 (9)
5	16 (18)
6	7 (8)
7	11 (12)
8	10 (11)

AIS - abbreviated injury scale, CT - computed tomography; ISS - Injury Severity Score; APACHE - Acute Physiology and Chronic Health Evaluation II; SOFA - Sequential Organ Failure Assessment; MAP - mean arterial blood pressure, tSAH - traumatic subarachnoid hemorrhage; WBC - white blood cell count; ABG - arterial blood gases; PaO_2_ - partial pressure of oxygen; PaCO_2_ - partial pressure of carbon dioxide; GFAP - glial fibrillary astrocytic protein: ICU - intensive care unit; LOS - length of stay; GOSE - Glasgow Outcome Scale-Extended. Results expressed as mean ± standard deviation; n (%) or median (interval interquartile).

On admission to the neuro-ICU, 71% (63) of patients required ventilatory support for a median of 6 (3 - 12) days. During neuro-ICU stay, 46% (41) of patients developed an infection, frequently around Day 5 (4 - 7). Respiratory tract infections occurred in 36% (32) of the patients, including 9 with ventilator-associated pneumonia and 23 with ventilator-associated tracheobronchitis. Other infections diagnosed in the neuro-ICU included catheter-associated urinary tract infection in 6% (5) and catheter-associated blood stream infection in 5% (4). The neuro-ICU length of stay (LOS) was 6 (4 - 15) days, and the total LOS was 11 (7 - 29) days. The in-hospital and 6-month mortality rates were 16% (14) and 28% (25), respectively. Unfavorable outcomes (GOSE score < 4) were documented in 48% (44) of the patients. The 6-month GOSE scores are included in table 1.

### Laboratory analysis

#### Pituitary axis

Thyroid-stimulating hormone, GH, and ACTH samples were available for 77 patients on admission, 73 patients on Day 3 and 47 patients on Day 7. Missing samples on Day 3 were related to 3 deaths and 1 transfer. By Day 7, 16 patients had been discharged, and 4 patients had been transferred. The median TSH level (IQR) values were as follows: admission = 0.63 (0.27 - 1.16) uU/mL, Day 3 = 0.67 (0.23 - 1.53) uU/mL, and Day 7 = 1.21 (0.70 - 1.98) uU/mL. The GH level median (IQR) values were as follows: admission = 173.53 (89.6 - 354.29) pg/mL, Day 3 = 240.95 (132.35 - 520.16) pg/mL, and Day 7 = 255.96 (148.10 - 510.88) pg/mL. Finally, the median ACTH level (IQR) values were as follows: admission = 1.3 (0.39 - 3.54), Day 3 = 2.35 (0.5 - 4.77), and Day 7 = 3.2 (1.3 - 6.72), as shown in table 2.

**Table 2:** Levels of anterior pituitary hormones

Hormone	Admission (n = 77)	Day 3 (n = 73)	Day 7 (n = 47)	p value
TSH (uU/mL)	0.63 (0.27 - 1.16)	0.67 (0.23 - 1.53)	1.21 (0.70 - 1.98)	0.001
GH (pg/mL)	173.53 (89 - 354)	240.95 (132 - 520)	255.96 (148 - 510)	0.003
ACTH (pg/mL)	1.3 (0.39 - 3.54)	2.35 (0.5 - 4.77)	3.2 (1.3 - 6.72)	0.002

TSH - thyroid stimulant hormone; GH - growth hormone; ACTH - adrenocorticotropic hormone. Ranges of reference: TSH 0.5 - 4.5uU/mL; GH: 400 - 10000pg/mL, ACTH 5 - 6pg/mL (range of reference from the ambulatory setting).

**Figure 1:**
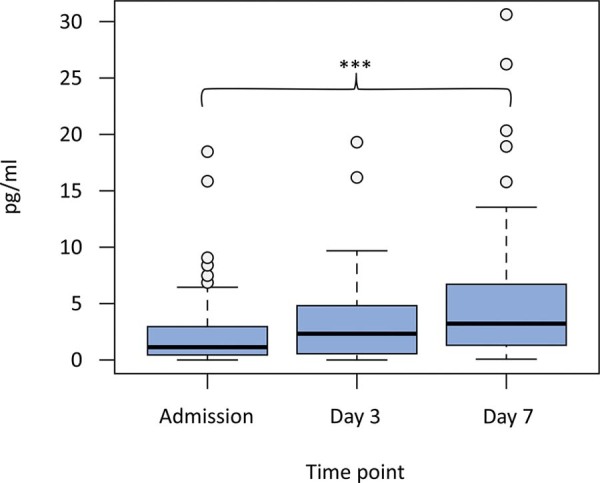
Adrenocorticotropic hormone levels after a traumatic brain injury. Figure displays the quantitative differences in adrenocorticotropic hormone levels on admission, Day 3 and Day 7 of the hospital stay. There was a significant difference in adrenocorticotropic hormone levels between admission and Day 7.

**Figure 2:**
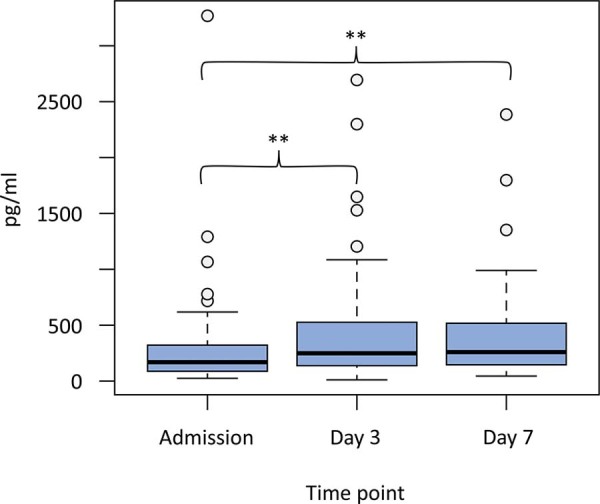
Growth hormone levels after a traumatic brain injury. Figure displaying the quantitative differences in growth hormone levels on admission, Day 3 and Day 7 of the hospital stay. The levels on admission were significantly lower than those on Day 3 and Day 7, but they were not different between Day 3 and Day 7.

**Figure 3:**
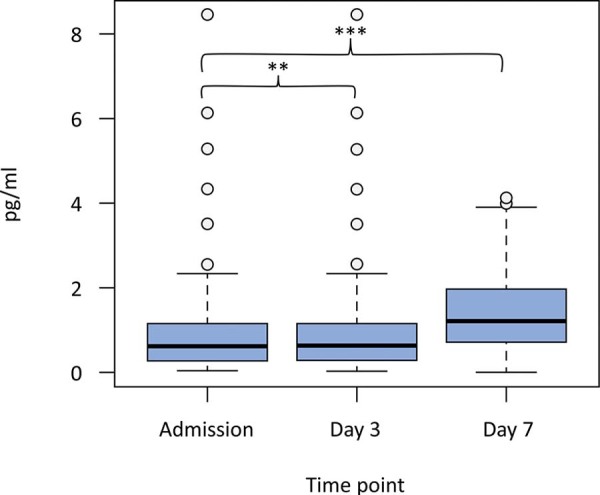
Thyroid stimulating hormone levels after a traumatic brain injury. Figure displaying the quantitative differences in thyroid stimulating hormone levels on admission, Day 3 and Day 7 of the hospital stay. Thyroid stimulating hormone levels on admission and Day 3 were lower than those on Day 7.

There was a significant difference between ACTH levels on admission and Day 7 [1.3 (0.39 - 3.54) *versus* 3.2 (1.3 - 6.72), p < 0.001] (Figure 1). In the case of GH, the levels at admission were significantly lower than those on Day 3 and Day 7 [173.53 (89 - 354) *versus* 240.95 (132 - 520), p = 0.007; 173.53 (89 - 354) *versus* 255.96 (148 - 510), p = 0.003] (Figure 2). Thyroid-stimulating hormone levels on admission and Day 3 were lower than those on Day 7 [0.63 (0.27 - 1.16) *versus* 1.21 (0.70 - 1.98), p = 0.003; 0.67 (0.23 - 1.53) *versus* 1.21 (0.70 - 1.98), p < 0.001] (Figure 3).

**Figure 4:**
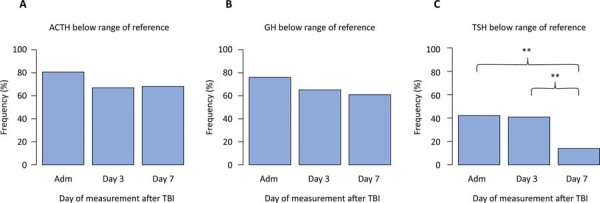
Frequencies of anterior pituitary hormones below the reference range at each time point. ACTH - adrenocorticotropic hormone; GH - growth hormone; TSH - thyroid-stimulating hormone; TBI - traumatic brain injury. There was a statistically significant difference in the frequency of lower thyroid-stimulating hormone levels between admission and Day 3 (p = 0.003) and between Day 3 and Day 7 (p = 0.004). Ranges of reference: thyroid-stimulating hormone 0.5 - 4.5uU/mL; growth hormone: 400 - 10,000pg/mL, adrenocorticotropic hormone: 5 - 60pg/mL (ranging from the ambulatory setting).

The proportion of patients with ACTH levels below the reference range (5 - 60pg/mL) was 81% (63/77) on admission, 75% (55/73) on Day 3, and 68% (32/47) on Day 7. There was no statistically significant difference in the frequency of low ACTH levels among the three time points [admission *versus* Day 7 (p = 0.4), admission *versus* Day 3 (p = 0.1), Day 3 *versus* Day 7 (p = 0.5)]. The proportion of patients with GH levels below the reference range (400 - 10,000pg/mL) was 76% (59/77) on admission, 65% (48/73) on Day 3, and 61% (29/47) on Day 7. No significant difference in the frequency of lower levels was found between the three time points (admission *versus* Day 3, p = 0.2; admission *versus* Day 7, p = 0.1; Day 3 *versus* Day 7, p = 0.7). There was only one case of GH above the reference range (10,237pg/mL), which was observed on Day 1. Finally, 42% (32/77) of patients had TSH levels below the reference range (0.5 - 4.5uU/mL) on admission, 41% (30/73) on Day 3, and 14% (7/40) on Day 7. There was a statistically significant difference in the frequency of lower TSH levels between admission and Day 3 (p = 0.003) and between Day 3 and Day 7 (p = 0.004) (Figure 4). In addition, 4% (3/77) of the patients had TSH levels above the reference range on admission, and no patients had TSH levels above the reference range on Day 3 or Day 7.

#### Logistic regression analysis

According to the linear regression analysis, there was a statistically significant direct association between admission ACTH levels and TBI severity, as assessed by the head AIS (p = 0.03), but not those on Day 3 (p = 0.7) or Day 7 (p = 0.06). Traumatic brain injury severity was also directly associated with GH levels on Day 7 (p = 0.03) but not on admission (p = 0.1) or Day 3 (p = 0.6). Finally, TBI severity was inversely associated with TSH levels on Day 3 (p = 0.03) but not on admission (p = 0.3) or Day 7 (p = 0.2). A positive association was found between GFAP and ACTH levels on Day 3 after trauma (OR 1.02, 95%CI 1.01 - 1.03, p < 0.001) but not on admission (p = 0.7) or Day 7 (p = 0.3). Additionally, there was a significant inverse association between GFAP and TSH levels on Day 7 after the injury (OR 1.02, 95%CI: 1.02 - 1.03; p = 0.04). When the severity of trauma, including other body regions (ISS), was considered, linear regression demonstrated an association between ISS and GH levels on Days 3 (p = 0.01) and 7 (p = 0.01). The mean arterial blood pressure (MAP) on admission was directly associated with the ACTH level on Day 3 (p < 0.001). Growth hormone (p = 0.3, p = 0.2, p = 0.4, respectively) and TSH (p = 0.5, p = 0.1, p = 0.1, respectively) levels were not associated with admission MAP. Glucose levels on admission were inversely associated with GH levels on Day 3 (p = 0.03).

There was a direct significant association between Day 1 ACTH levels and the ICU LOS (p = 0.014). Growth hormone levels on Day 3 were significantly associated with the number of days on the ventilator (p = 0.006) and hospital LOS (p = 0.0017). The logistic regression analysis revealed no significant associations between ACTH levels at any time point and in-hospital or 6-month mortality. GH levels were not associated with in-hospital or 6-month mortality. The same trend was observed between TSH levels and in-hospital mortality (p = 0.09, p = 0.5, p = 0.7) and 6-month mortality (p = 0.2, p = 0.9, p = 0.2). After dichotomizing the 6-month GOSE score into favorable (GOSE score > 4) or unfavorable (GOSE score < 4) outcomes, logistic regression revealed no significant associations between unfavorable outcomes and ACTH levels on admission (p = 0.23), Day 1 (p = 0.11) or Day 7 (p = 0.12). There was no significant association between unfavorable outcomes and GH levels at any study time point (p = 0.6, p = 0.8, p = 0.9) or between unfavorable outcomes and TSH levels (p = 0.3, p = 0.3, p = 0.3).

## DISCUSSION

Suppression of hormone levels was frequently documented upon admission, with a trend toward normalization within the first week. The levels of pituitary hormones within the first week after injury were associated with the severity of TBI, as measured by the head AIS, and with the levels of GFAP. In addition, when other variables were considered, GH was associated with the severity of overall trauma measured by the ISS, number of days on the ventilator, hospital LOS and serum glucose, whereas ACTH was associated with the ICU LOS and MAP. However, we did not find any significant associations between anterior pituitary hormone levels and other clinical outcomes, such as in-hospital and 6-month mortalities or 6-month functional deficits.

### Adrenocorticotropic hormones

Traumatic brain injury leads to acute activation of the hypothalamic–pituitary axis (HPA) as part of the stress response,^(41)^ initially through activation of the hypothalamic paraventricular nucleus (PVN), whose neurons secrete corticotropin-releasing hormone.^(42)^ The diagnosis of secondary adrenal insufficiency might be untenable in the acute setting of TBI; the insulin tolerance test is unfeasible in critically ill patients, and the corticotropin stimulation test evaluates mostly primary adrenal insufficiency without revealing the integrity of the entire HPA.^(43)^ However, ACTH and cortisol levels were evaluated simultaneously in a previous study by Cohan and colleagues, where transient secondary adrenal insufficiency was documented in approximately 50% of patients with TBI, mostly within the first 4 days. The adrenocorticotropic hormone level has been associated with a lower admission GCS score, lower blood pressure, and increased vasopressor usage but not with the 6-month functional outcome.^(44)^

Our study revealed lower levels of ACTH in a significant proportion of TBI patients within the first 24 hours after the injury. Although those levels increased progressively over time, they persisted below the reference values for up to 7 days. This might represent an impaired response to the injury that tends to improve. However, standardized values of reference in this setting have not been established, and multiple variables can affect these measurements in acutely ill patients in addition to the injury itself. Adrenocorticotropic hormone levels are directly associated with the severity of the clinical and serum biomarker manifestations of TBI, which might reflect a stronger stress response to the initial injury. We found no relationship with mortality or functionality, which is consistent with other studies.^(26,44)^ However, common scales used to assess disability, such as the GOS and GOSE, might be insufficient to measure more subtle variations in the quality of life in subjects with HPA dysfunction.^(45)^

### Growth hormone

Growth hormone is secreted by somatotroph cells located laterally in the anterior pituitary gland. Its main role during adulthood is to regulate metabolism, inducing the production of insulin-like growth factor I (IGF-I) in the liver.^(46,47)^ During acute periods of fasting and stress, GH secretion increases and stimulates lipolysis and the oxidation of free fatty acids, which decreases glucose and protein oxidation and, therefore, preserves protein and glycogen reserves.^(48)^

Growth hormone has been reported to be the most frequently diminished hormone in the acute^(26,49)^ and chronic phases of TBI.^(49)^ In the acute phase, GH levels are decreased in 50% of patients within the first week, which is associated with the severity of the injury.^(26,50)^ Although the consequences of GH deficiency in the acute stage after TBI and how it affects the response to the injury might not be clear, compelling evidence suggests that GH deficiency impacts long-term neuropsychological outcomes and quality of life.^(51)^ Our study revealed a high proportion of patients with decreased GH levels (76%) within the first 24 hours after a TBI, which remained diminished in the majority of patients through the first week (61%), with a slight tendency for improvement. Interpretation of the GH results in critically ill patients might be challenging given the multiple variables involved, including medical interventions and physiological response to systemic and local injuries.^(11,15)^ In addition, we did not measure IGF-I, which is the effector of GH in terms of glucose and lipid metabolism and maintains more stable plasma levels throughout the day than does GH. This is a limitation that hinders the interpretation of our results. Growth hormone levels were positively associated with the severity of both head trauma (head-AIS) and systemic trauma (ISS), which might be related to the response to the injury. In addition, serum glucose levels were inversely associated with GH levels, which might reflect the response of GH to fasting conditions. Growth hormone level was not associated with mortality or GOSE score.

### Thyroid-stimulating hormone

Thyroid-stimulating hormone is a glycoprotein produced in the anterior pituitary gland and constitutes the primary stimulus for thyroid hormone secretion.^(52)^ T3 and T4 increase the cellular metabolic rate and stimulate glycogenolysis, gluconeogenesis, and lipolysis.^(53,54)^ They also play pivotal roles in maintaining brain function and regulating the expression of genes responsible for cell differentiation, myelination, and neuroplasticity.^(55,56)^ The hypothalamic pituitary thyroid axis responds to multiple inputs, including inflammation and the nutritional state.^(57)^ Lower TSH, T4, and T3 levels have been described in patients with worse TBI outcomes.^(58,59)^ However, the available data are inconsistent.^(49,60)^ Secondary hypothyroidism occurs in approximately 20% of TBI patients within the first week of injury^(26)^ and in 0 - 20% of patients during the chronic phase,^(61,62)^ with a higher prevalence than in the general population.^(63)^

Thyroid-stimulating hormone was the least affected hormone in our cohort and showed a consistent increasing trend with each measurement, suggesting normalization in the early-postinjury period. It has been hypothesized that low frequencies of TSH alterations after TBI may be due to the anteromedial location of thyrotropic cells in the pituitary gland, as compared to hormones that are more frequently affected, such as GH, whose somatotropic cells are located in the lateral aspects of the gland and consequently are more susceptible to mechanical injury.^(10,64)^ However, this theory has not been confirmed. We found that the severity of injury caused by head AIS and the GFAP were inversely associated with the TSH level. Whether these findings could reflect metabolic adaptations to the injury or associated with direct injury and cell loss in the gland remains uncertain. We did not measure the levels of thyroid hormones, which could support our results; this is a limitation because it would have allowed a more comprehensive evaluation of thyroid function and made our results more comparable to those of previous studies that measured hormone levels. Lower levels of TSH could be associated with negative feedback from thyroid hormones; none of our patients had a history of hyperthyroidism. Our study did not find a significant association between TSH levels and hospital or 6-month mortality or functionality, as measured by the GOSE.

## CONCLUSION

This prospective traumatic brain injury study aimed to evaluate the dynamics of anterior pituitary hormones in the acute phase of injury and their relationship with clinical outcomes. Our results revealed significant alterations in anterior pituitary hormone levels, followed by a trend toward normalization during the first week. In addition, the results suggest an association between anterior pituitary hormone levels and the severity of traumatic brain injury but not with functionality. The interpretation of these findings warrants careful consideration owing to the complexities inherent in the critically ill patient population. The observed alterations in anterior pituitary hormone levels might be related to local brain injury and medical interventions, but they are also likely to be part of the adaptive response to metabolic stress in critically ill patients. Routine measurement of anterior pituitary function in the acute setting may not be cost-effective, considering the current evidence and unclear safety and benefits of treatment for these hormonal alterations. However, our results underscore the importance of close follow-up and attention to patients with clinical signs that might be secondary to pituitary disturbances, either in the acute phase of care or during recovery and rehabilitation.

Limitations of this study include the measurement of hormone levels at specific time points and the lack of measurements of other pituitary hormones for a complete profile. The measurement and interpretation of anterior pituitary function in this scenario are not standardized and can be influenced by medications, clinical conditions, and type of injury. In addition, the Glasgow Outcome Scale-Extended might not be an adequate tool to evaluate outcomes regarding neuroendocrine disorders; other methods that include specific symptoms, metabolic disturbances, and neuropsychological outcomes might yield more significant findings. Overall, this study highlights the need for increased awareness of the risk of pituitary dysfunction in the traumatic brain injury population and its potential long-term impact on quality of life and comorbidities. Although some guidelines recommend the measurement of pituitary function 3 - 6 months after traumatic brain injury, this is not a widespread routine practice in many centers.
